# Transforming the Patient Role to Achieve Better Outcomes Through a Patient Empowerment Program: A Randomized Wait-List Control Trial Protocol

**DOI:** 10.2196/resprot.5376

**Published:** 2016-04-21

**Authors:** Lisa Altshuler, Joseph Plaksin, Sondra Zabar, Andrew Wallach, Chester Sawicki, Sarita Kundrod, Adina Kalet

**Affiliations:** ^1^ Program on Medical Education, Innovations and Research (PrMEIR) Division of General Internal Medicine and Clinical Innovation New York University School of Medicine New York, NY United States; ^2^ Bellevue Hospital Center Division of General Internal Medicine and Clinical Innovation New York University School of Medicine New York, NY United States; ^3^ Department of Clinical Affairs and Affiliates New York University School of Medicine New York, NY United States; ^4^ Department of Psychology University of Minnesota Minneapolis, MN United States

**Keywords:** shared decision making, patient activation, health literacy

## Abstract

**Background:**

In the patient-centered medical home model of health care, both health care providers (HCPs) and patients must understand their respective roles and responsibilities, view the other as a partner, and use communication skills that promote shared decision making. This is particularly necessary in chronic conditions where outcomes depend on behavior change and in underserved populations where the burden of chronic disease is high.

**Objective:**

The objectives of this study are to determine if a Patient Empowerment Program (PEP) (1) is acceptable to patients and feasible across multiple clinical sites; (2) will increase patient preference for control in medical decision making, improve patient perceptions of patient-HCP communication, and increase patient activation; (3) is associated with an increase in diabetes self-management behaviors; and (4) has an effect on hemoglobin A _1c_(HbA _1c_) level.

**Methods:**

This study recruited English-speaking adult patients with type 2 diabetes mellitus from three urban clinical sites in New York City and randomized them to an immediate intervention group that completed the PEP intervention or a deferred intervention group that served as a wait-list control and completed the PEP intervention after 3-4 months. The PEP intervention consists of two facilitated small group sessions. Session 1 focuses on defining HCP and patient roles in the medical encounter by introducing ideal communication behaviors in each role and by providing both positive and negative examples of patient-HCP encounters. Session 2 focuses on practicing communication skills by role-playing with actors who serve as standardized health care providers. After the role play, participants set goals for their own health care and for future interactions with their HCPs. Outcome measures include the Patient Activation Measure; Ask, Understand, Remember Assessment; Krantz Health Opinion Survey; SF-12v2 Health Survey; Diabetes Self-Management Questionnaire; and HbA _1c_. These measures will be assessed at the time of enrollment, after the waiting period (deferred intervention only), and then postintervention at 1 week, 3 months, and 6 months.

**Results:**

Study recruitment occurred from November 2014 to June 2015, with a total of 80 patients enrolled. To date, 45 participants have attended at least one session of the PEP intervention. Further intervention sessions and post-intervention follow-up are ongoing, with data collection set to be completed in April 2016 and results of data analysis available by June 2016.

**Conclusions:**

From preliminary participant self-report data, our PEP intervention is acceptable to low-income, low–health literate patients and feasible to hold across multiple clinical sites. Participants have reported learning specific ways to change their behaviors at their next HCP visit (eg, stating their opinions, asking more questions). With the forthcoming quantitative data on participant attitudinal and behavior change, the PEP intervention may ultimately empower participants within the medical encounter and improve health outcomes.

## Introduction

### Background

More than 25.8 million Americans have type 2 diabetes mellitus (T2DM). In 2012, T2DM cost the United States $245 billion in both direct and indirect medical costs [ 1]. Comparable to the general population, it has been estimated that 10% of the New York City population suffers from T2DM, and patients with diabetes-related disorders occupy half of the hospital beds in the city [ 2]. The patient-centered medical home (PCMH) model, which strives to provide comprehensive care and improve patient’s self-management skills through increasing engagement with health care providers (HCPs), is ideally suited for T2DM care [ 3- 5].

Preparing patients for the PCMH model is challenging because of the inherent power differential between HCPs and patients. Efforts to prepare HCPs to practice in the PCMH model have included strategies for encouraging patient self-care activities and behavior change (eg, tailoring, brief negotiation, motivational interviewing) [ 6, 7]. Interventions have also been developed to educate patients about disease management and increase involvement in their care [ 8- 11]. However, both approaches fail to address the inherent asymmetry in the power dynamics of the physician-patient relationship [ 12- 21]. Increasing patient activation is one method that has been suggested to overcome these barriers. Activated patients are knowledgeable about their health conditions, confident in their ability to manage these conditions, and maintain their health by seeking information and performing health promoting behaviors [ 22]. In general, more activated patients, as determined by a higher score on the Patient Activation Measure (PAM) [ 22, 23], ask more questions during HCP visits [ 22- 24] and perform more self-management behaviors including diet and exercise [ 22, 23, 25- 28]. More specifically, patients with T2DM who have higher PAM scores report less difficulty in managing their diabetes than those with lower scores [ 28]. Furthermore, several interventions that increase patient activation have shown promise for improving outcomes [ 29- 32], most notably in congestive heart failure [ 29] and T2DM [ 30].

Standardized patient (SP) training, a well-established, performance-based intervention, offers a compelling method of activating patients to become partners in their health care. Standardized patients are trained to reliably and validly assess HCP clinical competence [ 33- 37] and, as a result, become more activated “real” patients who have higher expectations of HCPs and improved communication with HCPs [ 38- 43]. Additionally, SPs have improved their own health behaviors in terms of weight loss [ 44] as well as HIV testing and sexually transmitted disease prophylaxis [ 45]. More recently, these same training methods have been used to train standardized health care providers (SHPs) to assess communication between HCPs of different disciplines (eg, doctors and nurses) as part of interprofessional education [ 46- 48].

Our Patient Empowerment Program (PEP) seeks to adapt the successful SP methodology currently used in HCP education and translate it onto the patient side of the medical encounter [ 49]. To do so, the PEP incorporates ideas of shared decision making (SDM), involves role-playing, and helps patients develop the skills to give effective feedback to HCPs using validated checklists of observable behaviors. This intervention is both evidence based and theory supported and addresses the needs of multiple stakeholders, including health system quality leaders, HCPs, patient advocates, SP trainers, and patients. We focus on patients with T2DM because of its high prevalence in the population, the need for frequent contact with the health care system, and the numerous aspects of treatment (eg, lifestyle changes, medications, blood glucose monitoring, annual screenings, and so on) that patients must discuss with their HCPs.

### Objectives

The objectives of this study are to (1) assess the acceptability and feasibility of implementing the PEP across three urban clinical sites; (2) determine if a PEP will change patient preference for control in medical decision making, improve patient self-efficacy in patient-HCP communication, and increase patient activation; (3) determine if participation in the PEP is associated with an increase in diabetes self-management behaviors; and (4) explore the effect of the PEP on hemoglobin A _1c_(HbA _1c_) level.

## Methods

### Overview

The PEP is a randomized, wait-list control study (see Multimedia Appendix 1) that aims to enhance general communication skills so that patients can participate in SDM during office visits, become activated in their own health care, and better manage their medical conditions.

**Figure 1:**
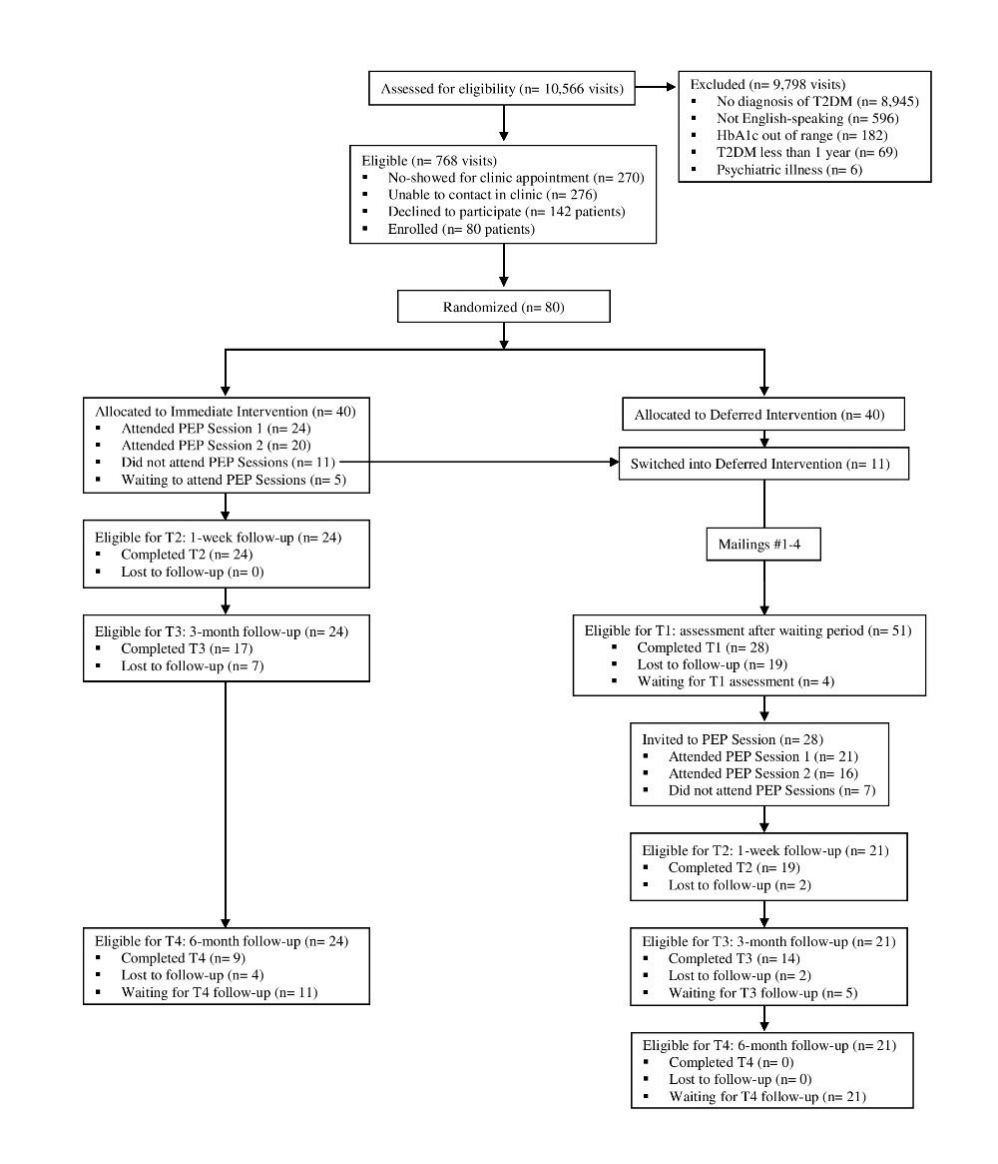
CONSORT flow diagram. HbA1c: hemoglobin A1c; PEP: Patient Empowerment Program; T2DM: type 2 diabetes mellitus.

Patients were recruited in cohorts of 20 and then randomly assigned to attend the PEP intervention immediately (immediate intervention) or after a waiting period (deferred intervention). All participants completed a baseline assessment at the time of enrollment (T0) and will complete follow-up assessments 1 week (T2), 3 months (T3), and 6 months (T4) after completion of the PEP intervention (see Figure 1). Additionally, the deferred intervention group was assessed at the end of the waiting period (T1), immediately before being invited to attend the PEP intervention. The T1 assessment for the deferred intervention group was approximately 3-4 months after randomization and was timed to coincide with the T3 assessment of the immediate intervention group. A wait-list control design was chosen to create a control group with multiple time points of data collection (deferred intervention T0-T1) while still allowing for all participants who enrolled in the study to complete the PEP intervention and post-PEP follow-ups.

Aside from attending the PEP intervention, all participants continued to receive their usual care, including all scheduled primary care and specialty clinic appointments, HbA _1c_monitoring, and all medications as directed by their physicians.

### Setting

Bellevue Hospital Center, Gouverneur Health, and Woodhull Medical Center are all within the Health and Hospitals Corporation, a large public hospital system in New York City. The adult ambulatory care clinics at these sites serve patients from the local community as well as immigrants from around the world, with English, Spanish, Mandarin, Cantonese, and Bengali among the most common languages spoken. Approximately one-third of patients have Medicaid or are without health insurance and the most common diagnoses in the clinic include obesity, hypertension, heart disease, diabetes, and asthma.

### Participants

We recruited patients with T2DM who presented to the adult ambulatory care clinic at each site. Patients eligible for inclusion in the study (1) were at least 18 years old, (2) had a diagnosis of T2DM for at least 1 year, and (3) their most recent HbA _1c_level was between 6.5% and 11%. Patients were excluded if they (1) were unable to speak English or (2) had a major psychiatric illness that impaired their ability to care for themselves (eg, schizophrenia, uncontrolled bipolar disorder, or uncontrolled depressive disorder).

### Recruitment, Randomization, and Retention

Research assistants (RAs) screened all of the clinic appointments each day to identify patients who were eligible for the study. Research assistants then briefly explained the study to all eligible patients who came to their appointment and obtained informed consent from those who decided to enroll (see Figure 1). After obtaining informed consent, RAs read participants a set of questionnaires to complete the initial (T0) assessment (see Table 1).

After 20 participants were enrolled in the study, a Web-based random number generator was used to generate a list of 20 integers between one and two. The list was refreshed until it contained 10 ones and 10 twos. Based on this list, participants were assigned in order of their study identification number to the immediate intervention (1) or deferred intervention (2) group. All participants in the immediate intervention group were contacted by phone to schedule the PEP intervention sessions and all participants in the deferred intervention group were contacted by phone and informed that they would be recontacted in 3-4 months to schedule their PEP intervention sessions.

Participants were compensated for participating in the study. They received US $10 and a MetroCard with US $10 at each of the two PEP intervention sessions. They also received US $60 and a MetroCard with US $10 for completing the study if they attended a post-PEP focus group.

In order to increase retention of deferred intervention participants, they received a letter from our program and an educational handout about T2DM in the mail approximately every 3 weeks. Handouts were sent in the same order and all handouts came from the Diabetes Care and Education website [ 50]. In total there were four mailings: (1) Nutrition to Help Manage your Diabetes and Weight, (2) Eating Healthy on a Lean Budget, (3) Know Your Blood Sugar Numbers, and (4) Managing and Preventing Hypoglycemia. All four of these handouts were short, only 1-2 pages in length, and contained general information about T2DM that participants should have already obtained from their HCPs as part of their routine treatment. Therefore, we do not expect that receiving these handouts will cause a significant difference in the two randomized groups or otherwise affect the results of the study.

### Outcome Measures

Outcome measures for the study primarily consist of self-report questionnaires assessing health attitudes and behaviors (see Table 1). Because of the low literacy of the patient population, RAs read all questionnaires to the participants and then recorded their responses. All questionnaires were asked in the same order and a standardized protocol was created so that all RAs asked and clarified questions in the same manner. Outcome measures were assessed at the time of enrollment (T0), after the waiting period (T1: deferred intervention group only), and postintervention after 1 week (T2), 3 months (T3), and 6 months (T4).

**Table 1:** Data collection.

Domain	Measures	Timing of assessment			
		Enrollment (T0)	After waiting period (T1) ( *deferred only*)	1 week after PEP ^a^ (T2)	3 and 6 months after PEP (T3, T4)
Demographics	Age, sex Race and ethnicity Education level Annual income	X			
Clinical characteristics	Number of years with diabetes Current diabetes treatment	X			
Health literacy	Newest Vital Sign [ 51- 53]	X			
Patient activation	Patient Activation Measure [ 23]	X	X	X	X
Ability to obtain information from HCPs ^a^	Ask, Understand, Remember Assessment [ 54]	X	X	X	X
Preference for control in medical decision making	Krantz Health Opinion Survey [ 55]	X	X	X	X
	Preference Control Scale [ 56]	X	X	X	X
Disease management	Diabetes Self-Management Questionnaire [ 57]	X	X		X
Health quality of life	SF-12v2 Health Survey [ 58]	X	X		X
Biological marker of disease severity	Hemoglobin A _1c_	X	X		X
PEP intervention feedback	Qualitative data analysis of focus group discussion			X	

^a^PEP: Patient Empowerment Program; HCP: health care provider.

#### Primary Outcome

The primary outcome of the study is patient activation, as measured by the short form of the PAM [ 23]. The PAM is a 13-item interval level, unidimensional, Guttman-like scale with four response options, ranging from 1 = disagree strongly to 4 = agree strongly. It has been validated across multiple patient populations and has been shown to be reliable, with Rasch person reliability estimates ranging from .73-.84. The overall score on the PAM ranges from 0-100 and PAM scores can be categorized into four levels of activation. In level one, the lowest level, patients believe taking an active role in their health is important but are unprepared for this role. In level two, patients have some knowledge but still struggle to manage their medical conditions. In level three, patients begin to take action in terms of self-management but do not have the skills to support or sustain their behavior. Finally, in level four, patients have adopted self-management behaviors and work on maintaining them in stressful life situations [ 23, 30].

#### Secondary Outcomes

Secondary outcomes of the study include the ability to obtain information from HCPs, preferences for information and control in medical decision making, health quality of life, and diabetes self-care behaviors.

The ability of participants to obtain health information from HCPs was assessed by the Ask, Understand, Remember Assessment (AURA) [ 54]. The AURA is a 4-item interval level scale with four response options, ranging from 1 = disagree a lot to 4 = agree a lot. It is strongly correlated with chronic disease self-efficacy ( *r*= .31) and moderately correlated with disease knowledge ( *r*= .11). It also has good internal consistency reliability, with Cronbach’s alpha = .75.

Participants’ preferences for information and control in medical decision making were assessed by the Krantz Health Opinion Survey (Krantz) [ 55] and the Preference Control Scale (PCS) [ 56]. The Krantz [ 55] is a 16-item dichotomous (agree/disagree) multidimensional scale containing two subscales: Information and Behavioral Involvement. The Information subscale measures the desire for health information and the Behavioral Involvement subscale measures the desire to engage in health behaviors. The Krantz is moderately correlated ( *r*= .31) with an established health locus of control scale, and test-retest reliability was .74 for the Information subscale, .71 for the Behavioral Involvement subscale, and .59 for the overall scale. The PCS [ 56] is a 1-item Likert-type interval level scale with five response options. Choices range from “I prefer to make the decision about which treatment I will receive” to “I prefer to leave all decisions regarding treatment to my doctor.”

Participants’ health quality of life was measured by the SF-12v2 Health Survey [ 58], a 12-item interval level, multidimensional scale containing a Physical Component summary score and Mental Component summary score. The SF-12v2 Health Survey has been validated against other physical and mental health scales with Spearman correlation coefficients for each item ρ = .32-.61. It also has high internal consistency reliability (Mosier alpha = .78-.88) and moderate-high test-retest reliability (Physical Component ICC = .78, Mental Component ICC = .60).

Finally, participants’ diabetes-specific health behaviors were assessed using the Diabetes Self-Management Questionnaire (DSMQ) [ 57]. The DSMQ is a 16-item interval level scale with four response options that range from 0 = does not apply to me to 3 = applies to me very much. It includes a Summary Scale as well as four subscales: Glucose Management, Dietary Control, Physical Activity, and Health-Care Use. The DSMQ was validated against a longer diabetes self-care scale and has been shown to significantly correlate with HbA _1c_. It also has good internal consistency reliability, with Cronbach’s alpha = .60-.84.

#### Exploratory Outcome

HbA _1c_will serve as an exploratory outcome for the study. HbA _1c_is exploratory for this study because participants did not undergo HbA _1c_testing at specific times during the study period. Rather, participants will continue to have HbA _1c_levels monitored as part of their routine care and the HbA _1c_closest to enrollment (T0) and each follow-up time point (T1, T3, and T4) will be collected from the electronic medical record (EMR) and used for data analysis. Therefore, it is possible that a significant number of follow-up HbA _1c_values may be missing or not collected at the time of follow-up and those that are will not correlate precisely to the same time period as the questionnaires.

#### Potential Confounding Variables

Previous studies have shown that there are numerous confounding variables that affect patients’ ability to participate in SDM [ 15, 17, 18, 59]. In order to account for these variables, demographic information was collected at enrollment (T0). Demographics included age, sex, race/ethnicity, educational attainment, and income level. Clinical characteristics included the number of years diagnosed with diabetes and current diabetes treatment (eg, lifestyle modifications, oral medications, insulin, or both oral medication and insulin).

In the study population, health literacy is also expected to be a significant confounding variable. Health literacy was measured at enrollment (T0) using the Newest Vital Sign (NVS) [ 51- 53]. The NVS is a food label accompanied by 6 questions that are scored dichotomously (correct/incorrect). It has been validated against a longer health literacy questionnaire with an area under the receiver operating characteristic curve of .88. A score of less than 2 has a sensitivity of 72% and a specificity of 87% for predicting low health literacy, while a score of less than 4 has a sensitivity and specificity of 100% and 64%, respectively. The NVS also has good internal consistency reliability, with Cronbach’s alpha = .76.

#### Acceptability and Feasibility of the Intervention

Acceptability and feasibility were judged based on (1) willingness of potential research subjects to enroll in the study, (2) responses to the activities during the PEP sessions, and (3) ability to engage in the role-playing scenarios in PEP session 2. After completion of the PEP intervention, all participants were invited to attend a focus group to discuss their experiences with and reactions to the PEP intervention, which were then audiotaped and transcribed. The transcribed text will be parsed into segments that represent a perspective or theme and each segment will then be independently coded by three readers. Through an iterative process among the coders, a single parsimonious coding scheme will be derived and then applied to all transcripts by the same three coders toward providing a thematic analysis of the data.

### Intervention: Patient Empowerment Program

Our PEP is a two-session course led by a clinical health psychologist (LA) and assisted by research staff (JP, SK, and CS). Each session is 2 hours in length and the two sessions are held approximately 1 week apart. The PEP intervention is designed to be a group experience, with 2-6 participants attending each session. Ideally, the same participants who attended session 1 will return for session 2, but because of the limited availability of both research staff as well as participants, it is expected that this may not always be the case.

#### PEP Intervention Development

The PEP intervention curriculum (see Table 2) and materials for each PEP session were developed with input from patients as well as HCPs across all levels of training. Input was obtained via focus groups, all led by a clinical health psychologist (LA). Six focus groups for patients with T2DM (n=26) were held across all three study sites. In these groups, patients discussed their experiences with HCPs, difficulties managing diabetes, and opinions about participating in the PEP. This information was used to create content for video clips of patient-HCP interactions and cases for role-playing scenarios with SHPs. Overwhelmingly, patients reported that they thought it would be beneficial to participate in a program like PEP [ 60].

Focus groups were also held with internal medicine attending physicians (n=11), primary care residents (n=16), and medical students with at least 1 year experience on clinical rotations (n=11). In these groups, HCPs discussed their experiences with patients and expectations for patient engagement/activation. Overwhelmingly, HCPs reported preferring patients who were more informed about their medical conditions, who raised questions or concerns during the office visits, and who performed self-management behaviors in between appointments to those who were less activated [ 61].

After these focus groups were completed and intervention materials were created, 6 patients from the focus groups were invited to complete a beta test of the PEP intervention. During the beta test, they provided feedback about timing of the sessions, realism of the video clips, and feasibility of asking patients to complete a 10-minute role-play scenario with SHPs. Their feedback was used to revise all materials before recruitment for the PEP intervention began.

**Table 2:** Patient Empowerment Program intervention curriculum.

	Session 1		Session 2	
Session Element	Part 1	Part 2	Part 1	Part 2
Time	1 hour	1 hour	1 hour	1 hour
Task	Learn disease-specific case & HCP ^a^checklist	Use checklist to rate video interactions	Practice case with SHPs ^a^	Plan to apply model to own health care
Goal	Set standards for high-quality HCP and activated patient behaviors	Understand range of provider behaviors Learn to accurately describe provider behavior	Experience models of physician-patient interactions Shift social dynamic by empowering patient	Increase patient activation
Target	Learn standards for HCP behaviors: listen, ask questions, develop shared goals Learn standards for patient behaviors: share information, make choices, negotiate with provider	Learn to recognize elements of good/poor shared decision making and communication Practice describing HCP and patient behavior	Learn to be an active partner in health care encounters Give constructive feedback to providers	Reflect on lessons learned Set individualized goals for own health care and interaction with HCP
Theory	Develop cognitive framework for health care interactions	Vicarious learning of interpersonal skills in exam room	Behavioral rehearsal Experiential learning	Behavioral intentions

^a^HCP: health care provider; SHP: standardized healthcare provider

#### Intervention Standardization and Fidelity

In order to standardize the intervention, a curriculum and detailed manual were created. A group of 7 actors with previous SP experience were trained to play SHPs at the beginning of the study and reoriented to each case by an SP trainer before each intervention session. Throughout the intervention sessions, participants were asked to rate each activity to determine if the learning goals were being achieved, using a simple visual Likert scale with responses ranging from 0 = not at all to 3 = very much. All PEP intervention sessions were also audiotaped so that they could be transcribed to evaluate consistency across each run of the intervention.

#### Patient Empowerment Program Session 1

**Figure 2:**
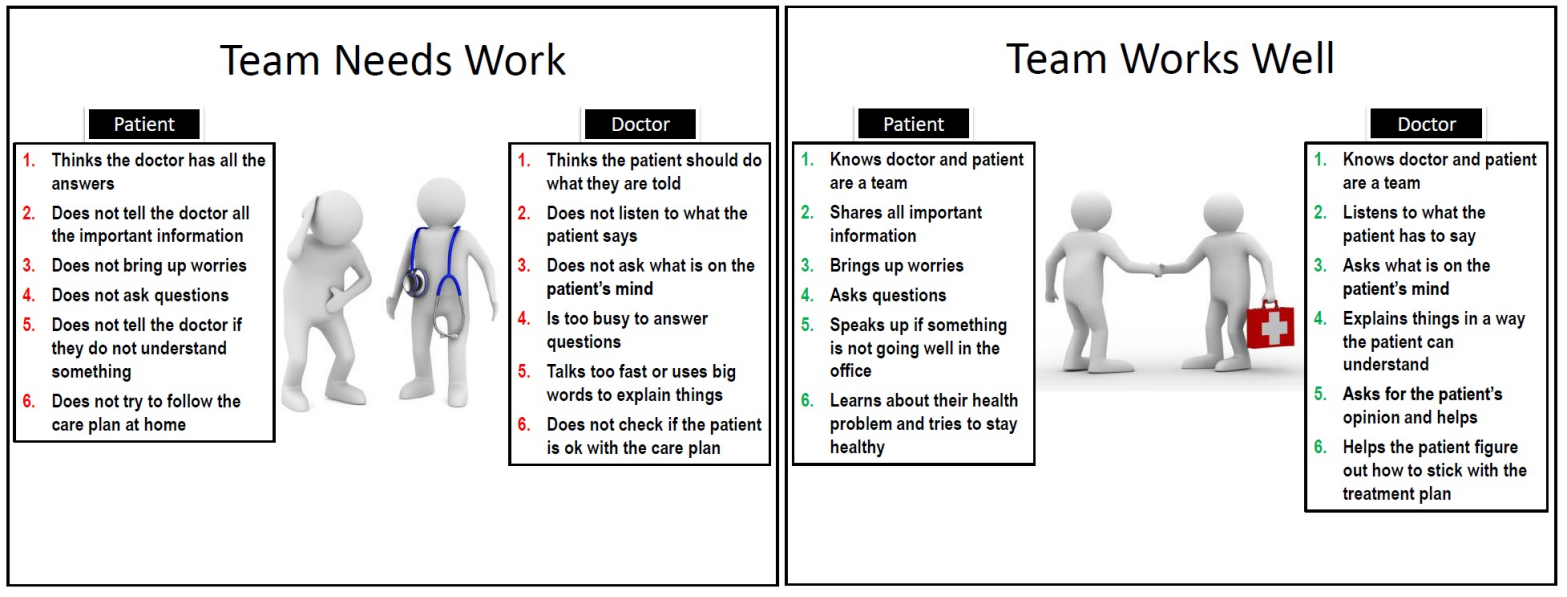
Framework for patientÃƒÆ’Ã†â€™Ãƒâ€šÃ‚Â¢ÃƒÆ’Ã‚Â¢ÃƒÂ¢Ã¢â€šÂ¬Ã…Â¡Ãƒâ€šÃ‚Â¬ÃƒÆ’Ã‚Â¢ÃƒÂ¢Ã¢â‚¬Å¡Ã‚Â¬Ãƒâ€¦Ã¢â‚¬Å“health care provider interactions.

In the beginning of PEP session 1, participants discussed their experiences with HCPs and beliefs about the role of the patient and HCP in the medical encounter. Two posters were used to frame this discussion (see Figure 2), which describe specific behaviors of an effective patient-HCP team (ie, Team Works Well) and one that is not effective (ie, Team Needs Work). This framework was used to anchor discussions of the video clips and role-playing scenarios throughout the remainder of the PEP intervention.

Participants then watched three pairs of video clips of patient-HCP interactions. These video clips were created using actors who were experienced SPs and to represent a range of sex, race, and ethnicity for both the patient and HCP. Each pair of video clips represented a different aspect of a conversation between a patient and HCP, including (1) HCP taking the patient's medical history, (2) HCP providing information, and (3) decision making about the treatment plan. All visits were designed as follow-up visits, implying an ongoing relationship between patient and HCP within the interactions.

The first video in each pair represented a negative example of communication. In these videos, the patients were passive and did not voice their perspective or engage actively with the HCP. The HCPs, while trying to be helpful and sympathetic, were rushed, followed their own agendas, and acted with some sense that they knew the best approach. After the video clip, participants rated both the patient’s and HCP’s communication based on behaviors listed on the Team Works Well poster and discussed what each party could have done to improve the communication and the quality of the visit.

The second video in each pair was example of positive communication in the same situation. The patients in these videos were more activated and engaged with their HCPs, while the HCPs demonstrated a patient-centered approach. Similar to the first video, afterwards participants rated the patient and HCP and then had a brief discussion about what was different or improved.

#### Patient Empowerment Program Session 2

PEP session 2 gave participants the opportunity to practice the communication skills that were discussed in session 1. Participants were first split into pairs to complete a brief role-play scenario with SHPs. While one participant was role-playing, the other observed and rated both the participant and the SHP in real time. The observing participant then led a debriefing of the scenario in order to practice giving feedback. After the debriefing, the participants switched places and the second participant completed a different role-play scenario.

In the second part of PEP session 2, participants applied what they learned to an individualized 10-minute role-play scenario with a SHP, created specifically for each participant based on self-reported difficulties with diabetes. Common topics for this scenario included struggles with adhering to a healthy diet, difficulties performing frequent finger sticks to monitor blood glucose, and overall frustration when trying to control blood sugar numbers. Research personnel observed these role-plays and led a debriefing afterward. PEP session 2 concluded with a group discussion about lessons learned from the role-plays and each participant worked with a member of the research staff to create an individualized action plan for both diabetes self-care and future medical encounters. All ideas for the action plan were participant generated, but research staff provided feedback on the feasibility of their goals and brought up potential barriers to those goals when participants were unable to identify any on their own.

#### Post-PEP Focus Group

Approximately 1 week after the completion of PEP session 2 all participants were invited to attend a focus group. The goals of focus group were to (1) collect the first set of follow-up questionnaires, (2) gather qualitative data on intervention, and (3) get feedback on each element of the PEP curriculum.

### Follow-Up

After completion of the PEP intervention, participants will be followed up for 6 months and will complete repeat assessments at 1 week (T2), 3 months (T3), and 6 months (T4) after the PEP intervention (see Table 1). The T2 assessment will take place either at the beginning of the focus group, to avoid any bias introduced by discussing the PEP intervention again, or by telephone for any participants who do not come to the focus group. The T3 and T4 follow-ups will occur by telephone and any concurrent HbA _1c_available at these times will be collected from the EMR.

### Sample Size

Prior studies have pilot-tested interventions to increase patient activation and found that PAM scores increased by a range of 4-8 points [ 30, 31]. This difference was statistically significant, is thought to be clinically significant, and translates to a moderate effect size, Cohen’s *d*=0.48. Given the repeated-measures design of the study, setting alpha = .05 and beta = .2, and using the effect size calculated from the literature, the required sample size will be n=36 patients. It is our goal to have 40 participants complete the PEP intervention.

In anticipation of significant participant dropout over the course of the study, we aimed to enroll 40 patients from Bellevue Hospital Center, 20 from Gouverneur Health, and 20 from Woodhull Medical Center. From these patients, it was our goal that 20 from Bellevue Hospital Center and 10 each from Gouverneur Health and Woodhull Medical Center would complete the PEP intervention. Because of these concerns about dropout, all participants who were randomized to the immediate intervention group but unable to attend the PEP intervention at that time were contacted for the T1 assessment with the deferred intervention group and invited back to attend the PEP intervention as part of that group (see Figure 1).

### Data Analysis

First, descriptive statistics including mean, standard deviation, range, skewness, and kurtosis for all continuous variables as well as frequencies for all categorical variables will be calculated at each time point (T0-T4). Because of concerns about the relatively small sample size and low literacy/health literacy, the normality of all continuous variables will be assessed both graphically with histograms as well as statistically using the Shapiro-Wilk and Kolmogorov-Smirnov tests, and transformations will be made if necessary. Then we will assess for any baseline (T0) differences between two separate groups: (1) the randomly determined immediate intervention and deferred intervention groups and (2) all participants who attended the PEP intervention and those who did not. Both of these analyses will be conducted using chi-square test for categorical variables and either independent samples *t*test or Wilcoxon rank sum test for continuous variables.

Next, analyses will be conducted within each intervention group to evaluate for any effect of the PEP intervention on the primary, secondary, and exploratory outcomes using chi-square test for categorical variables (eg, PAM level, PCS) and either repeated-measures *t*test or Wilcoxon signed rank test for continuous variables (eg, PAM score, AURA, Krantz, SF-12v2 Health Survey, DSMQ, and HbA _1c_). Initial assessment (T0) will be compared with each of the post-PEP assessments (T2-T4) for each variable. For the deferred intervention group, initial assessment (T0) will be compared with the assessment at the end of the waiting period (T1), which will then be compared with each post-PEP assessment (T2-T4).

After analyzing each group individually, the groups will be combined in order to have a larger sample size for pre- and post-PEP comparisons. Pre-PEP intervention data for these analyses will consist of data from enrollment (T0) for participants in the immediate intervention group and data collected at the end of the waiting period (T1) for participants in the deferred intervention group. Repeated-measures analyses will be conducted on the primary, secondary, and exploratory outcomes as described above and pre-PEP data (either T0 or T1) will be compared with each post-PEP assessment (T2-T3).

Finally, multivariate analyses will be conducted using a series of generalized mixed-effects models to determine what effect patient characteristics, group membership, and time have on each continuous outcome measure. In each model, the dependent variable will be the outcome measure (eg, PAM, AURA, Krantz, SF-12v2 Health Survey, DSMQ, and HbA _1c_). Independent variables will include participant characteristics such as age, sex, race/ethnicity, education level, annual income, number of years with T2DM, and health literacy as measured by the NVS. Other independent variables will include group (immediate vs deferred intervention) and time (T0-T4).

## Results

Recruitment for this study began in November 2014. As of June 2015, we met our enrollment goal of 80 patients. However, because of differences in site policies, the distribution of enrolled participants does not match our initial goals. By site, we have enrolled 40 participants from Bellevue Hospital Center, 31 from Gouverneur Health, and 9 from Woodhull Medical Center. As Woodhull Medical Center is located in a different part of New York City and serves a slightly different patient population, we plan to return to that site within the next few months to recruit an additional 11 patients.

Out of these 80 participants enrolled, the 71 from Bellevue Hospital Center and Gouverneur Health have been invited to attend the PEP intervention. Because of staff turnover and site-specific policies, we have as of yet been unable to hold any PEP intervention sessions at Woodhull Medical Center. Of the 71 participants invited to attend the PEP intervention, 45 have attended PEP session 1, 36 have attended the full PEP intervention, and 33 have returned for the post-PEP focus group. Of the 11 participants switched from the immediate intervention group to the deferred intervention group, 6 completed the T1 assessment and 3 attended at least one session of the PEP intervention. Coding and thematic analysis of the focus group discussions is not completed, but participants have reported that the PEP intervention was not only acceptable but a valuable experience that made them feel more empowered in their own health care. Several participants have even reported seeing their HCPs after completing the PEP intervention and changing their behaviors in the visit, which resulted in improvement in the quality of their visit.

Follow-up data collection is still underway and all patients who attended at least PEP session 1 will be called for all follow-up data points. Follow-up data collection is scheduled to conclude in April 2016 and the results of our data analysis are expected to be available by June 2016.

## Discussion

Although there have been significant shifts in the power hierarchy between HCPs and patients, there continues to be an asymmetry that makes it difficult for patients to engage in true SDM as envisioned by the PCMH model. Little is understood about how to best activate patients to make the most of their visits with HCPs. With input from both patients and HCPs, we have developed an innovative PEP that seeks to prepare patients with T2DM to be better partners in their care and collaborate effectively with HCPs. We focused on improving patients’ communication skills in the context of the medical encounter by discussing behavioral descriptors for activated patients and then using performance-based training, adapted from SP methodology, to develop these skills.

Study enrollment and implementation of the PEP intervention have been shown to be feasible across multiple hospital sites and acceptable to patients. Participants were able to engage in the skills development tasks and to self-reflect on their experiences with HCPs. Initial qualitative responses from participants were positive and collection of quantitative post-PEP data will conclude over the next few months, which will allow us to assess the effect of this intervention on patient activation and self-reported measures of diabetes care.

## Appendix

Multimedia Appendix 1 CONSORT-EHEALTH checklist V1.6.2 [ 62].

## Abbreviations

AURA: Ask, Understand, Remember Assessment
DSMQ: Diabetes Self-Management Questionnaire
EMR: electronic medical record
HbA1c: hemoglobin A1c
HCP: health care provider
Krantz: Krantz Health Opinion Survey
NVS: Newest Vital Sign
PAM: Patient Activation Measure
PCMH: patient-centered medical home
PCS: Preference Control Scale
PEP: Patient Empowerment Program
RA: research assistant
SDM: shared decision-making
SHP: standardized health care provider
SP: standardized patient
T2DM: type 2 diabetes mellitus
